# The Prevalence of Ocular Allergy and Comorbidities in Chinese School Children in Shanghai

**DOI:** 10.1155/2017/7190987

**Published:** 2017-08-21

**Authors:** Yanqing Feng, Xiangning Wang, Fang Wang, Rongming Liu, Lu Chen, Shuqin Wu, Xia Yang, Miaoying Chen, Yu-Qing Rao, Jing Li

**Affiliations:** ^1^Department of Ophthalmology, Shanghai Kongjiang Hospital, Shanghai, China; ^2^Department of Ophthalmology, The Sixth People's Hospital Affiliated to Shanghai Jiao Tong University School of Medicine, 600 Yishan Road, Shanghai 200233, China; ^3^Department of Moral Education, Institute for Advanced Study of Teachers, Bureau of Education of Yangpu District, Shanghai, China; ^4^Erlian Primary School of Yangpu District, Shanghai, China; ^5^Department of Ophthalmology, Xin Hua Hospital Affiliated to Shanghai Jiao Tong University School of Medicine, Shanghai, China

## Abstract

**Objective:**

To investigate the prevalence and features of ocular allergy (OA) and comorbidities among school children in Shanghai, China.

**Methods:**

This was a population-based cross-sectional study. Each participant completed an ISAAC-based questionnaire. The prevalence of OA symptoms, allergic rhinitis (AR) asthma, atopic dermatitis (AD), and sensitization to mites, pollen, and food was analyzed.

**Results:**

A total of 724 and 942 completed questionnaires from the 7–9-year-old (young group) and the 12–14-year-old (teen group) groups were analyzed, respectively. The overall prevalence of OA symptoms was 28%. However, more young students (10.6%) reported mild to severe daily life interference caused by OA than the teens (5.7%). The young group had higher prevalence of diagnosed allergic conjunctivitis (10.2%). The overall prevalence of AR symptom, diagnosed asthma, and diagnosed AD was 40.4%, 11.6%, and 16.7%, respectively. Young children had higher prevalence of diagnosed AR and AD than the teens. There were gender associated differences in the prevalence of AR and asthma among young children, but not among the teens. The comorbidities associated with OA was also analyzed. Sensitization to mites, food, and pollen was associated with higher prevalence of allergic conditions.

**Conclusions:**

OA together with other allergic conditions affected a significant number of children in Shanghai.

## 1. Introduction

Ocular allergy (OA) is frequently associated with other allergic conditions such as rhinitis and asthma and treated as an associated symptom with nasal allergy [1, 2]. For this reason, the prevalence and characterizations of OA are often not surveyed individually and subsequently the data was scarce. Among reported studies on the prevalence of OA, the definition of the condition, the method used, and the targeted population of investigation varied significantly [3–8]. These differences made estimating the prevalence of OA in general population and comparison among different regions and ethnic groups very difficult [9, 10]. A unified definition of OA and standardized methods of survey would be useful in order to generate comparable data on the epidemiology of OA worldwide.

The International Study on Asthma and Allergies in Childhoods (ISAAC) had developed effective methods to survey the prevalence of asthma, allergic rhinitis, and eczema among children [11]. The questionnaires designed by ISAAC have been tested and proved by studies from almost every region in the world in many languages including Chinese. Unfortunately, OA was not included in the ISAAC study. However, attempts had been made to adopt questionnaires and survey methods of ISAAC to investigate the prevalence of OA and comparable data was generated among children from different regions [3, 5, 7].

China is one of the largest countries in the world with almost 20% of the world population and a land mass close to the entire continent of Europe. There are vast differences in climate and environment across the country. However, research on the epidemiology of allergic diseases in China was limited [12]. In the last century, the reported overall prevalence of common allergic conditions was low in China [1]. This has changed rapidly in the recent two decades. For example, in Phase III of study of ISAAC, the reported prevalence of rhinitis ever, rhinitis, and rhinoconjunctivitis among children of 13-14 years in Beijing was at 46.1%, 35.6%, and 10%, respectively, reflecting the annual increase of 0.30% to 0.66% for these conditions [13]. The causes for such significant increase in allergic conditions among Chinese are likely multifactorial, including changes of the environment, life style, childhood nutrition, and disease awareness. More studies using the same or similar protocols are needed to fully understand the profile of allergic conditions in China.

Despite the fact that there were several epidemiological studies on asthma, allergic rhinoconjunctivitis, and atopic dermatitis (AD) among children in China [14–19], the prevalence of OA remains unknown. In this study, we adopted the core questions developed by ISAAC study to determine the prevalence of OA and other common allergic conditions in school children at the ages of 7–9 and 12–14 years in metropolitan Shanghai, China.

## 2. Methods

### 2.1. Study Cohorts and Ethnics

This was a cross-sectional study conducted between March 2016 and May 2016 in Yang Pu District, Shanghai, China. A pilot study on about 350 students between ages of 7 and 12 years was carried out in 2014. The pilot study included questionnaires designed based on ISAAC questionnaires and on-site ophthalmic examinations for ocular allergy symptoms. The questionnaire was given to students two days before on-site ophthalmic examination and was collected on the day of examination. More questions than given in this study were included in the pilot questionnaire and the students' answers were carefully analyzed to determine the optimal number of questions and the proper phrasing of each question.

Based on the pilot study, we estimated that the prevalence of OA symptoms among primary school students was around 25%. Therefore a sample size of 626 was required in order to reach a confidence level of 0.95 and a precision of 0.05 with assumed sensitivity of 0.8 and specificity of 0.9. Assume a total of 10% of dropout rate, we estimated that 700 students in each targeted age group were needed.

The schools participated in this study were randomly picked by the Bureau of Education of Yangpu District. From four primary schools, 950 students between grades 1 and 3 were enrolled for the 7–9-year-old group. From five middle schools, 1050 students between grades 6 and 8 were enrolled for the 12–14-year-old group. Only completed questionnaires of children within the desired age range with consent were analyzed.

This study was performed in accordance with the Declaration of Helsinki (1964) and approved by the Ethics Review Boards of the Administration of Education and School, Shanghai (YPJY20151202), and Kong Jiang Hospital, Shanghai (KJ20151201), China. Informed written consent was obtained from the parents or guardians of all participated children.

### 2.2. Questionnaire

**Figure 1:**
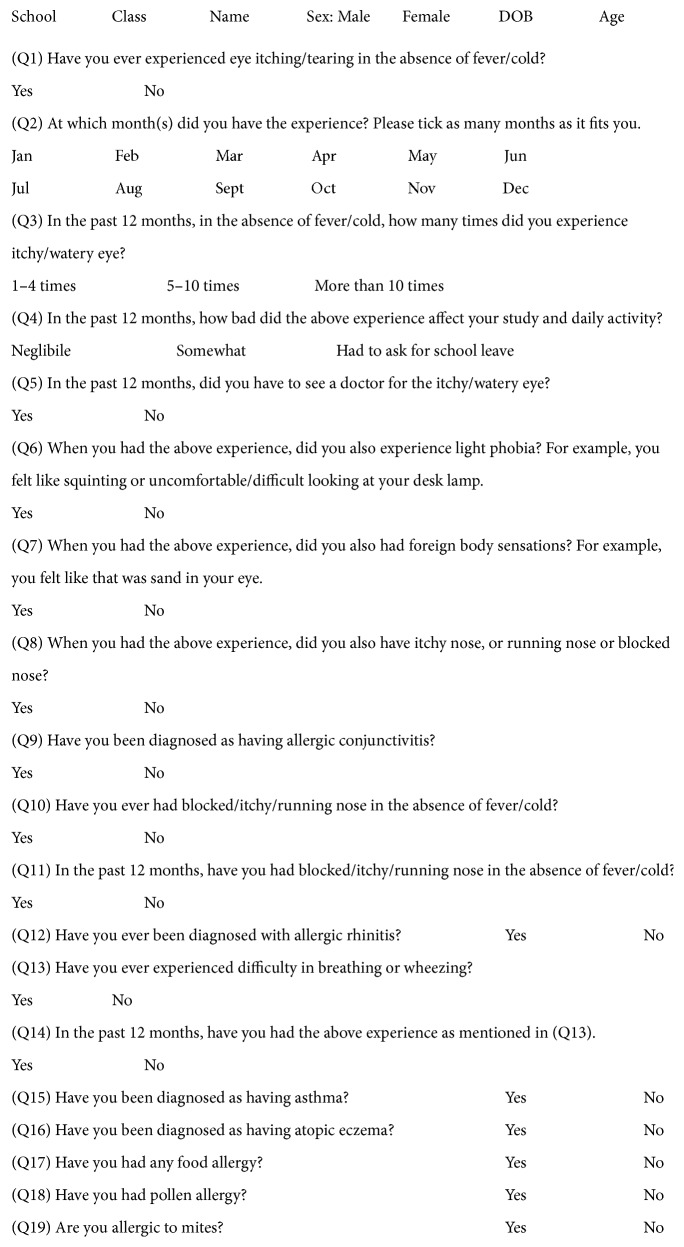
The English version of the questionnaire used in this study. This questionnaire was designed based on ISAAC.

Based on the pilot study, we designed a multiple choice questionnaire which was essentially based on the International Study of Asthma and Allergies in Childhood (ISAAC) [11]. The English translation of the questionnaire was provided (Figure 1). The questionnaire was distributed to each student of the participated classes together with the consent form and an information letter to the parents/guardians by the class monitor. Students were told to take it home and fill it out together with their parents/guardians if they agreed to participate into the study. It was collected on the following day together with the consent form.

### 2.3. Definition of OA and Other Allergic Conditions

A positive OA symptom was defined if a child had previous history of itchy/watery eye and experienced at least 1 episode of such experience in the past 12 months. Seasonal OA was defined if the symptoms occurred in two or more consecutive months. Perennial OA was defined if the symptoms occurred in four or more months. A positive allergic rhinitis symptom was concluded if a child had previous and recent (within 12 months) experiences of blocked/itchy nose in the absence of fever/cold. A positive rhinoconjunctivitis symptom was concluded if a child had itchy/running/blocked nose together with itchy/watery eyes. A positive asthma symptom was concluded if a child had previous and recent (within 12 months) experiences of short of breath/wheezing. Note that separate questions were asked whether they had been diagnosed with rhinitis or asthma.

### 2.4. Statistical Analysis

Statistical analysis was performed using Statistical Package for the Social Sciences (SPSS) Version 19 (IBM Corporation, Armonk, NY). Statistical significance was accepted at *p* ≤ 0.05. Chi-square test was performed to evaluate the association between different variables.

## 3. Results

### 3.1. Basic Demographic Information of the Participated Children

A total of 2000 questionnaires were distributed to 4 primary schools and 5 secondary schools and 1820 were completed with consent. Among these, 154 were excluded because the age of these students was not in the desired range. The basic demographic information of the qualified children were provided in Table 1. In total, we had 724 qualified questionnaires from the group of 7–9 years old and 963 qualified questionnaires for the group of 12–14 years old.

### 3.2. Prevalence and Features of OA Symptoms

The age and gender stratified prevalence of OA symptoms were given in Table 2. Overall about 28.0% of all children experienced eye tearing and itching. This percentage was slightly higher in the young group (29.6%) than the teen group (27.0%), but the difference was not statistically significant. Ocular foreign body sensation was more frequently reported than light phobia in both groups. About 7% of the children had all OA symptoms investigated: tearing, itching, foreign body sensation, and light phobia. There was no statistically significant differences between the young and teen groups and among boys and girls of the same age group regarding the prevalence and symptoms of OA.

The severity and characteristics of OA symptoms were summarized in Table 3. In both groups, about 19.6% of the children reported the OA symptoms for less than 5 times in the past 12 months. The OA symptoms were seasonal in about 6.1% of the children, and perennial in about 11.3% of the children. There were no differences in the frequency and seasonality of OA among children of different gender or age.

However, there was significant differences regarding the interference of OA to daily life among children of different age and gender. Higher percentage of children at the young age reported that OA interfered with their daily activity. Consistently, higher percentage of young children sought medical help and were diagnosed with allergic conjunctivitis. Between boys and girls, more boys in the young age sought medical help and diagnosed with allergic conjunctivitis. However, these gender differences disappeared in the teen group.

### 3.3. Prevalence of Allergic Rhinitis, Asthma, and Atopic Dermatitis and the Coexistence of These Conditions with Ocular Allergy

We also investigated the prevalence of AR symptoms, diagnosed AR, diagnosed asthma, and diagnosed AD among these children (Table 4). The prevalence of AR symptoms was higher than OA, at 40.4% overall with no differences between young and teen groups. However, there was significant decrease in the prevalence of diagnosed AR in the teen group compared to the young group. The overall prevalence of diagnosed asthma was 11.6% among all children with no difference between young group (13.1%) and teens (10.4%). However, in both groups, the prevalence of diagnosed asthma was significantly lower in girls than in boys. Diagnosed AD occurred at 16.7% among all children. Again, the young group showed significantly higher prevalence (27.2%) than the teen group (8.7%).

Among all children with OA symptoms, there were 18 boys and 26 girls in the young group and 34 boys and 35 girls in the teen group who reported no other allergic symptoms investigated here. This accounted for 6.1% and 7.3% of the young and teen group, respectively.

### 3.4. Concurrence of OA Symptoms with Other Allergic Conditions

The coexistence of AR, asthma, and AD with OA was listed in Table 5. In both young and teen groups, the most common coexisting allergic condition was allergic rhinitis, at about 61% among all children studied, followed by asthma at 29% and atopic dermatitis at 25%. The percentages of children with coexisting allergic rhinitis and atopic dermatitis were higher in the young group than in the teen group. But the percentage of coexisting asthma was similar between the two groups. More boys tended to have coexisting atopic dermatitis (48.3%) and diagnosed asthma (24.1%) than girls (33.7% and 13.3%, resp.) in the young group, but the difference disappeared in the teen group. The percentage of children with coexisting asthma was similar between the two age groups.

**Figure 2:**
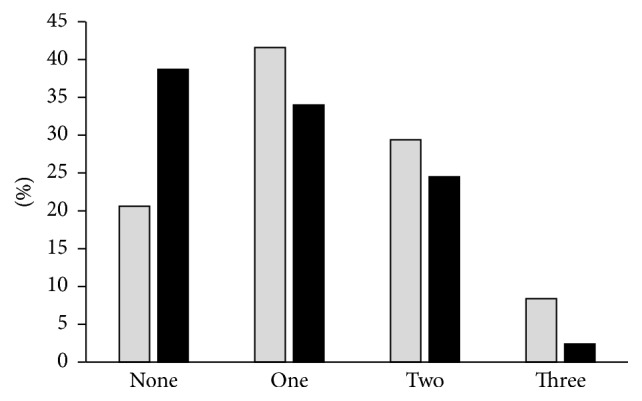
The percentage of children with none to three coexisting allergic conditions (allergic rhinitis, asthma, and atopic dermatitis) within the young group (grey columns) and the teen group (black columns) who had OA symptoms.

Overall, children at the young age had higher prevalence of allergic conditions (Figure 2). For example, the percentage of children with none of the above allergic conditions was 20.6% in the young group and 38.7% in the teen group. Furthermore, more children in the young group (about 8.4%) had coexisting of three or more allergic conditions than that in the teen group (about 2.4%).

### 3.5. Prevalence of Sensitization to Mite, Food, and Pollen and Its Association with OA

We also investigated the prevalence of sensitization to three general allergens: mite, pollen, and food (Table 6). Our results showed mite allergy was most common (overall 17.6%), followed by pollen (overall 12.8%) and food allergy (overall 12.3%). The percentage of mite allergy decreased significantly in the teen group compared to young group. There was also a significant difference in the percentage of food allergy between boys and girls in the teen group.

The prevalence of OA, allergic rhinitis, asthma, and atopic dermatitis was analyzed among children with allergy to mites, food, and pollen (Table 7). Children with allergy to any of these allergens had higher prevalence of allergic conditions than those without (Tables 7 and 4). In general, the sensitization to these allergens causes more allergic conditions in young children than in the teens. For example, OA and AD prevalence was significantly higher among young children with mite, food, and pollen allergy. Allergic rhinitis was most frequently associated in children with sensitization to these allergens, followed by OA and AD.

## 4. Discussion and Conclusions

There is a general paucity of data on the prevalence of OA in general population worldwide. As far as we were aware, there was no report on the prevalence of OA among mainland Chinese children. Here we adopted the ISAAC questionnaires to investigate the prevalence of OA symptoms and other allergic conditions among school children in metropolitan Shanghai, China. We chose two age groups in order to discern possible age-related changes. Our study revealed that close to 30% of school children in metropolitan Shanghai experienced OA symptoms. In Phase I of study of ISAAC, about 10.1% of 6-7-year-old children in Shanghai had itchy eyes [1]. Therefore our study confirmed that there was a rapid increase of children with OA symptoms among children in Shanghai. This percentage was higher than what was reported in a similar study among Brazilian children aged 12–18 years, which was at 20% [7]. The prevalence of OA in the teen group, which were at the age of 12–14 years, was 27% in this study. It was close to a telephone survey of itchy/red eyes among the US households which reported a 34% prevalence of OA symptoms in children [8].

Although there were no age or gender differences in the prevalence of OA symptoms, the frequency of tearing/itching episode, or seasonality of the symptom, our data suggested that the children of young age were more sensitive to OA than the teens. For example, about 10% of the children in the young group and 6% of the children in the teen group reported moderate to severe interference to daily activities due to OA. This corresponds to 36.0% and 21.4% of the children with OA in the young and teen groups, respectively. In a similar study mentioned above, 30.5% of the children at the age of 12–18 years reported severe symptoms [7]. A close analysis of the data revealed that almost all the children who reported moderate to severe daily life interference sought medical help. This explained the differences in the percentage of children seeking medical help within the two groups. We believe that it also contributed to the higher percentage of young children diagnosed as having AC (10.2%) than the teens (2.4%). However, these differences did not necessary reflect differences in the graveness of the condition. On the contrary, we believe that possible reasons for the reduced percentage reporting daily life interference and the need for medical help among teens were that the children become more tolerant to the symptoms as they grow older, and also they were busier in school and had less time to see a doctor than the young ones.

The overall prevalence of AR, diagnosed AR, asthma, and AD found in the study was very similar to what was reported by other groups also using ISAAC study protocol among Chinese children [12, 20]. The ISAAC Phase III study reported that the prevalence of rhinitis ever and rhinitis among children of 13-14 years old in Beijing was at 46.1% and 35.6%, respectively [13]. However, Shanghai was not included in Phase III of the study of ISAAC. Our results compensated for the lack of data. Furthermore, we found that the prevalence of diagnosed AR and AD was lower in the teen group than in the young group. Similar results were reported in other studies among Chinese children [21–23]. Again we confirmed the overall increase of allergic conditions in Shanghai over the last decades, suggesting that the changes in environment, nutrition, and life style had significant impact on the prevalence of allergic conditions.

The most common comorbidity of OA is AR. In pediatric outpatient clinic, the comorbidity of AR in children with allergic conjunctivitis was reported to be as high as 97% [24]. This number was lower in general population [25]. We found that about 67.8% of the young children and 54.4% of the teens with OA had AR symptoms. The coexistence of diagnosed AR and AD was also in lower percentage among the teen group than among the young group. Consistently, higher percentage of children in the teen group had only OA symptoms. This could due to the fact that less children in the teen group reported diagnosed AR and AD. Because this was a cross-sectional study, we were not able to discern whether OA tended to become a single condition as children grow older.

A gender difference in the prevalence of allergic conditions was also observed in this study, especially in the young group. Boys at the young age had reported higher percentage of having diagnosed AC, AR symptoms, diagnosed AR, and diagnosed asthma. These differences largely reduced and disappeared in the teen group. While similar observations were made by other studies, the reasons for such differences remained unknown [26–28].

Many factors trigger allergic reaction. In this study, we investigated the prevalence of sensitization to mite, food, and pollen among these children. Sensitization to mite was the most common, especially among young children. However, the percentage of children who were sensitive to mite decreased significantly in the teen group, a phenomena also reported by other groups [29, 30]. At the meantime, the prevalence of food and pollen sensitization remained similar between the two groups. Overall, children with sensitization to these allergens had higher prevalence of allergic conditions, especially in the young group. Therefore our results suggest that more attention should be given to young children with the above sensitization to avoid the development of allergic conditions.

This study has several limitations. First of all, we conducted the survey in the spring time which was a peak allergy season locally. This may result in more acute recognition and memory of allergic reactions, especially for ocular allergy and allergic rhinitis, leading to a higher prevalence of these conditions. Secondly, since it was a descriptive, self-reported study, the correct understanding and interpretation of each question and the accurate recollection of the participating students/guardians about their health conditions were critical to the reliability of the results. For example, different child may have different standard for “experience affect your study and daily activity.” Children/parents in the teen group may also forget more about medical history than the teen group. Face-to-face interview and on-site examination by a medical professional would have improved the quality of data significantly. Thirdly, as a cross-sectional study, we were not able to dissect the cause and consequence between various observations. A long-term follow-up on these children would give us more insightful data on the profile of allergic conditions among these children.

In conclusion, our study showed that ocular allergy, together with other allergic conditions, affected a significant number of school children in Shanghai, especially among primary school students. Adequate recognition of these conditions by children, parents, school, and medical professionals is needed. Preventative measures and prompt treatment of the symptoms should be recommended to the public and school administrations. These included effective vacuuming of the household, avoiding going to highly pollenated areas, more selective food, and a constant supply of nonprescriptive antihistamine medications at school.

## Figures and Tables

**Table 1:** Basic demographic information of the participants.

Age	Number of children		
	Boys	Girls	Total
7 years	129	114	243
8 years	141	136	277
9 years	102	102	204
Subtotal	372	352	724
12 years	175	184	359
13 years	178	189	367
14 years	106	110	216
Subtotal	459	483	942
Grand total	831	835	1666

**Table 2:** Prevalence of ocular allergy symptoms stratified by age and gender.

	Number of children (% in the group)								*χ* ^2^	*p*
	Young (*n* = 724)				Teen (*n* = 942)					
	Boys	Girls	*χ* ^2^	*p*	Boys	Girls	*χ* ^2^	*p*		
	(*n* = 372)	(*n* = 352)			(*n* = 459)	(*n* = 483)				
Tearing/itching	117 (31.5%)	97 (27.6%)	1.318	0.255	117 (25.5%)	132 (27.3%)	0.409	0.555	1.992	0.168
FB sensation	45 (12.1%)	45 (12.8%)	0.078	0.822	72 (15.7%)	76 (15.7%)	0.000	1.000	3.597	0.066
Light phobia	34 (9.1%)	31 (8.8%)	0.025	0.897	46 (10.0%)	60 (12.4%)	1.358	0.258	2.300	0.143
Tearing/itching with FB sensation	45 (12.1%)	45 (12.8%)	0.078	0.822	72 (15.7%)	76 (15.7%)	0.000	1.000	3.597	0.066
Tearing/itching with light phobia	34 (9.1%)	31 (8.8%)	0.025	0.897	45 (9.8%)	60 (12.4%)	1.629	0.215	2.101	0.165
Tearing/itching with light phobia and FB sensation	22 (5.9%)	22 (6.2%)	0.036	0.877	31 (6.8%)	40 (8.3%)	0.788	0.390	1.358	0.284

Pearson Chi-square analysis was performed and the *p* values were obtained from 2-sided exact significance tests. The *χ*^2^ and *p* values listed within each age group were the results of comparing the numbers of affected boys and girls within the group, thus reflecting the gender differences within age group. The *χ*^2^ and *p* values of the last two columns were the results of comparing the total number of affected children between young and teen groups. FB: foreign body.

**Table 3:** Severity and features of ocular allergy symptoms stratified by age and gender.

	Number of children (% in the group)								*χ* ^2^	*p*
	Young (*n* = 724)				Teen (*n* = 942)					
	Boys	Girls	*χ* ^2^	*p*	Boys	Girls	*χ* ^2^	*p*		
	(*n* = 372)	(*n* = 352)			(*n* = 459)	(*n* = 483)				
Tearing/itching frequency									1.970	0.579
1–4 times	84 (22.6%)	68 (19.3%)	1.109	0.775	87 (19.0%)	87 (18.0%)	1.438	0.487		
5–10 times	18 (4.8%)	17 (4.8%)			22 (4.8%)	26 (5.4%)				
More than 10 times	14 (3.8%)	12 (3.4%)			12 (2.6%)	19 (3.9%)				
Seasonality									1.656	0.437
Seasonal	21 (5.6%)	20 (5.7%)	0.006	0.997	28 (6.1%)	33 (6.8%)	2.093	0.351		
Perennial	46 (12.4%)	43 (12.2%)			42 (9.2%)	57 (11.8%)				
Daily life interference									15.595	0.001
No	73 (19.6%)	63 (17.9%)	4.284	0.232	98 (21.3%)	103 (21.3%)	2.066	0.559		
Moderate	38 (10.2%)	35 (9.9%)			23 (5.0%)	27 (5.6%)				
Severe	4 (1.1%)	0 (0%)			2 (0.4%)	2 (0.4%)				
Diagnosed AC	49 (13.2%)	25 (7.1%)	7.261	0.010	12 (2.6%)	12 (2.5%)	0.016	1.000	43.536	<0.001
Medical help	52 (14.0%)	28 (8.0%)	6.610	0.012	21 (4.6%)	22 (4.6%)	0.000	1.000	25.262	<0.000

Pearson Chi-square analysis was performed and the *p* values were obtained from 2-sided exact significance tests. The *χ*^2^ and *p* values listed within each age group were the results of comparing the numbers of affected boys and girls within the group, thus reflecting the gender differences within age group. The *χ*^2^ and *p* values of the last two columns were the results of comparing the total number of affected children between young and teen groups. AC: allergic conjunctivitis.

**Table 4:** Prevalence of other allergic symptoms as stratified by age and gender.

	Number of children (% in the group)								*χ* ^2^	*p*
	Young (*n* = 724)				Teen (*n* = 942)					
	Boys	Girls	*χ* ^2^	*p*	Boys	Girls	*χ* ^2^	*p*		
	(*n* = 372)	(*n* = 352)			(*n* = 459)	(*n* = 483)				
AR symptoms	162 (43.5%)	127 (36.1%)	4.207	0.041	173 (37.7%)	211 (43.7%)	3.502	0.064	0.122	0.763
Diagnosed AR	95 (25.5%)	65 (18.5%)	5.254	0.025	87 (19.0%)	81 (16.8%)	0.766	0.395	4.710	0.034
Diagnosed asthma	61 (16.4%)	34 (9.7%)	7.205	0.008	57 (12.4%)	41 (8.5%)	3.899	0.055	2.953	0.090
Diagnosed AD	111 (29.8%)	86 (24.4%)	2.670	0.113	34 (7.4%)	48 (9.9%)	1.896	0.203	100.5	<0.001

Pearson Chi-square analysis was performed and the *p* values were obtained from 2-sided exact significance tests. The *χ*^2^ and *p* values listed within each age group were the results of comparing the numbers of affected boys and girls within the group, thus reflecting the gender differences within age group. The *χ*^2^ and *p* values of the last two columns were the results of comparing the total number of affected children between young and teen groups. AR: allergic rhinitis. AD: atopic dermatitis.

**Table 5:** Coexistence of other allergic conditions among children with OA symptoms.

	Number of children (% in the group)								*χ* ^2^	*p*
	Young (*n* = 214)				Teen (*n* = 252)					
	Boys	Girls	*χ* ^2^	*p*	Boys	Girls	*χ* ^2^	*p*		
	(*n* = 116)	(*n* = 98)			(*n* = 120)	(*n* = 132)				
AR symptoms	83 (71.6%)	62 (63.3%)	1.670	0.240	62 (51.7%)	75 (56.8%)	0.672	0.448	8.911	0.003
Diagnosed AR	52 (44.8%)	39 (39.8%)	0.550	0.490	30 (25.0%)	32 (24.2%)	0.019	1.000	17.364	<0.001
Diagnosed AD	56 (48.3%)	33 (33.7%)	4.663	0.037	10 (8.3%)	17 (12.9%)	1.358	0.309	59.939	<0.001
Diagnosed asthma	28 (24.1%)	13 (13.3%)	4.054	0.055	19 (15.8%)	19 (14.4%)	0.102	0.860	1.622	0.219

Pearson Chi-square analysis was performed and the *p* values were obtained from 2-sided exact significance tests. The *χ*^2^ and *p* values listed within each age group were the results of comparing the numbers of affected boys and girls within the group, thus reflecting the gender differences within age group. The *χ*^2^ and *p* values of the last two columns were the results of comparing the total number of affected children between young and teen groups. AR: allergic rhinitis. AD: atopic dermatitis.

**Table 6:** Prevalence of sensitization to mite, pollen, and food as stratified by age and gender.

	Number of children (% in the group)								*χ* ^2^	*p*
	Young (*n* = 724)				Teen (*n* = 942)					
	Boys	Girls	*χ* ^2^	*p*	Boys	Girls	*χ* ^2^	*p*		
	(*n* = 372)	(*n* = 352)			(*n* = 459)	(*n* = 483)				
Mite allergy	94 (25.3%)	77 (20.5%)	1.155	0.295	68 (14.8%)	55 (11.4%)	2.436	0.123	31.420	<0.001
Food allergy	55 (14.8%)	45 (12.8%)	0.608	0.452	39 (8.5%)	66 (13.7%)	6.346	0.013	2.696	0.114
Pollen allergy	60 (16.1%)	44 (12.5%)	1.963	0.170	53 (11.5%)	56 (11.6%)	0.001	1.00	2.865	0.103

Pearson Chi-square analysis was performed and the *p* values were obtained from 2-sided exact significance tests. The *χ*^2^ and *p* values listed within each age group were the results of comparing the numbers of affected boys and girls within the group, thus reflecting the gender differences within age group. The *χ*^2^ and *p* values of the last two columns were the results of comparing the total number of affected children between young and teen groups.

**Table 7:** Prevalence of allergic conditions among children with sensitization to mite, food, and pollen.

	Mite (*n* = 294)				Food (*n* = 205)				Pollen (*n* = 213)			
	Young	Teen	*χ* ^2^	*p*	Young	Teen	*χ* ^2^	*p*	Young	Teen	*χ* ^2^	*p*
	(*n* = 171)	(*n* = 123)			(*n* = 100)	(*n* = 105)			(*n* = 104)	(*n* = 109)		
OA symptoms	94 (55.0%)	52 (42.3%)	4.612	0.034	55 (55.0%)	36 (34.3%)	8.903	0.003	62 (59.6%)	43 (39.4%)	8.659	0.004
AR symptoms	118 (69.0%)	73 (59.3%)	2.931	0.107	59 (59.0%)	58 (55.2%)	0.296	0.672	73 (70.2%)	57 (52.3%)	7.169	0.008
Diagnosed AR	98 (57.3%)	65 (52.8%)	0.577	0.477	45 (45.0%)	33 (31.4%)	4.002	0.061	57 (54.8%)	48 (44.0%)	2.470	0.132
Diagnosed asthma	49 (28.7%)	45 (36.6%)	2.069	0.164	35 (35.0%)	26 (24.8%)	2.569	0.127	33 (31.7%)	29 (26.6%)	0.677	0.452
Diagnosed AD	80 (46.8%)	22 (17.9%)	26.367	<0.001	57 (57.0%)	17 (16.2%)	36.980	<0.001	53 (51.0%)	15 (13.8%)	33.888	<0.001

Pearson Chi-square analysis was performed and the *p* values were obtained from 2-sided exact significance tests. The *χ*^2^ and *p* values listed within each sensitizer were the results of comparing the numbers of children between the group, thus reflecting the age differences.
